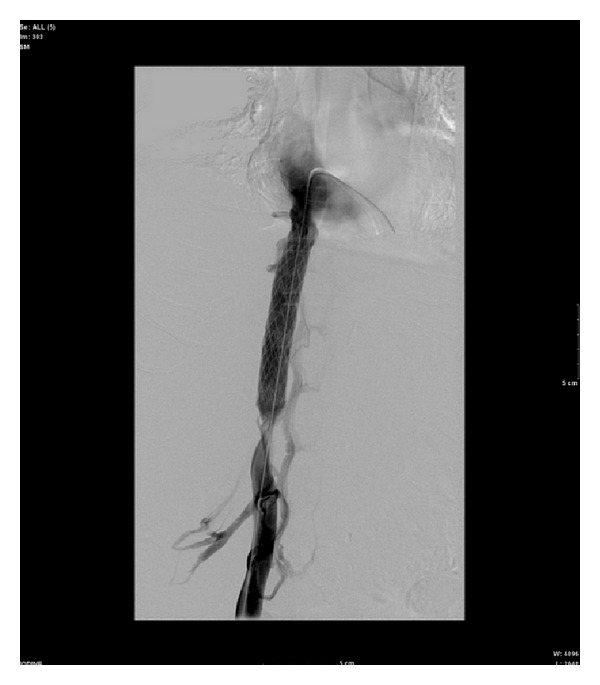# Inferior Vena Cava Torsion and Stenosis Complicated by Compressive Pericaval Regional Ascites following Orthotopic Liver Transplantation

**DOI:** 10.1155/2013/576092

**Published:** 2013-12-10

**Authors:** Adam Alli, Richard Gilroy, Philip Johnson

**Affiliations:** Department of Interventional Radiology and Gastroenterology, University of Kansas Medical Center, 3901 Rainbow Blvd., Kansas City, KS 66160, USA

## Abstract

Inferior vena cava (IVC) stenosis and torsion are well-described rare complications following orthotopic liver transplantation (OLT). We present a case of inferior vena cava intermittent torsion and stenosis complicated by compressive regional ascites. To the best of our knowledge, this is the second case of post-OLT regional ascites related compressive IVC stenosis reported and the first reported case of torsion complicated by regional ascites compression.

## 1. Introduction

We report the case of inferior vena cava (IVC) torsion following orthotopic liver transplantation (OLT) complicated by compression secondary to compressive regional ascites. To the best of our knowledge, this is the second case of post-OLT ascites related compressive IVC stenosis reported and the first reported case of torsion complicated by regional ascites compression.

## 2. Case Report

A 51-year-old male with Hepatitis C cirrhosis and hepatocellular carcinoma underwent piggyback orthotopic liver transplantation (OLT). During the immediate postoperative period, the patient had typical recovery with postoperative sonographic interrogation demonstrating expected perioperative edema. Increased velocities were reported near the inferior vena and portal veins which resolved with improving perioperative edema. No additional vascular compromise was identified in the immediate postoperative period.

The patient presented 4 weeks following OLT with shortness of breath and was found to have bilateral pleural effusions, recurrent ascites, and acute renal insufficiency. The findings were all assumed to be related to OLT vascular compromise. Thoracentesis and paracentesis (3 liters of ascitic fluid drained) were performed, and the following day ultrasound and Doppler imaging were performed to evaluate the hepatic vasculature. This revealed mild ascites without evidence of hepatic vascular compromise. Clinically, the patient demonstrated no improvement and had additional paracentesis 3 days following admission in which 5 liters of ascitic fluid was drained. On the 10th day following admission, laparoscopic exploration of the abdomen with liver biopsy and lysis of adhesions was performed. The liver biopsy revealed sinusoidal congestion without evidence of hepatic graft rejection or malignancy. On the 13th day following admission, repeat ultrasound with Doppler imaging demonstrated severe stenosis of the intrahepatic vena cava, portal hypertension, and perihepatic ascites. The stenosis was shown to be worse in the supine position.

**Figure 1:**
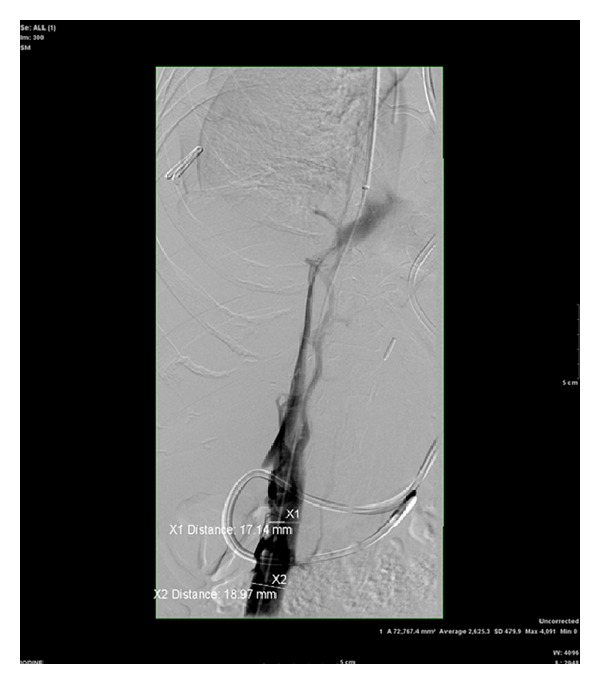


The following day, inferior vena cavagram was performed demonstrating high grade intrahepatic IVC stenosis with extensive collateral flow through the azygous system and thoracolumbar venous plexus (Figure 1).

**Figure 2:**
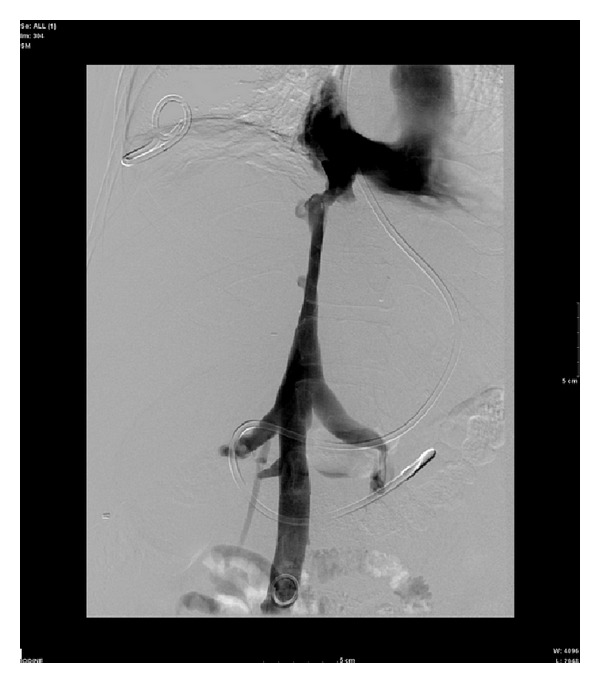


The pressure gradient across the stenosis measured 11 mm Hg. The stenosis was sequentially dilated with a 12 mm × 40 mm Conquest balloon (Conquest angioplasty balloon: C. R. Bard, Inc.) and a 16 mm × 40 mm Atlas balloon (Atlas angioplasty balloon: C. R. Bard, Inc.). Postangioplasty venography each time demonstrated no significant improvement in stenosis. The stenosis was then angioplastied with an 18 mm × 40 mm XXL balloon (XXL angioplasty balloon: Boston Scientific Corporation). Postangioplasty venography demonstrated moderate improvement in the stenosis (Figure 2).

The pressure gradient remained 11 mm Hg. At this point, the etiology of the stenosis was unknown and reevaluation with cross-sectional imaging was favored prior to stenting. Computed tomography (CT) of the abdomen demonstrated fluid surrounding the intrahepatic IVC. The fluid was percutaneously drained.

**Figure 3:**
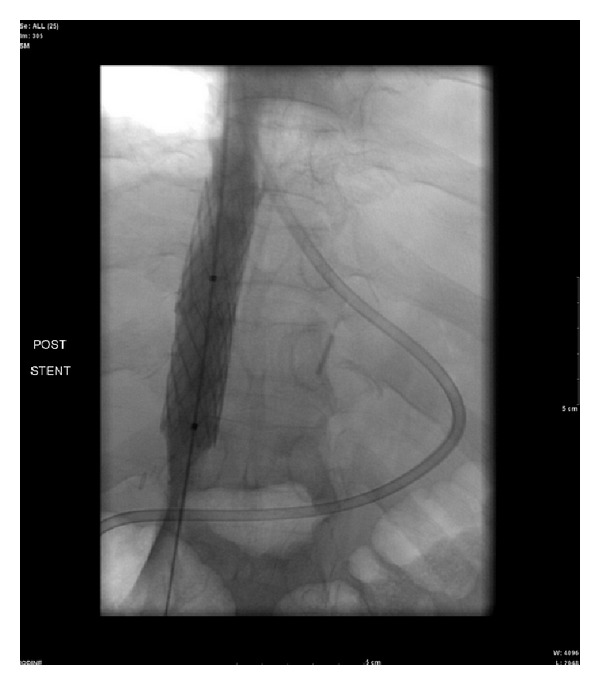


The patient returned for additional inferior vena cavagram and evaluation on the 23rd day following admission. Venogram demonstrated high grade intrahepatic IVC stenosis with pressure gradient across the stenosis measuring 15 mm Hg. A 4010 Palmaz stent (Palmaz stent: Cordis Corporation) was mounted on a 16 mm × 40 mm angioplasty balloon and the stent was positioned at the level of the inferior vena cava stenosis and deployed (Figure 3).

Completion angiography demonstrated excellent angiographic result within the stent; however, there continued to be a stenosis within the intrahepatic portion of the inferior vena cava below the stent. Therefore, a second 4010 Palmaz stent was mounted on a 16 mm × 40 mm angioplasty balloon and deployed. Both stents were fully deployed and there was excellent flow through the stents on completion angiography. However, there was some moderate narrowing of the IVC below the stents. Additionally, there was no significant change in pressure gradient.

**Figure 4:**
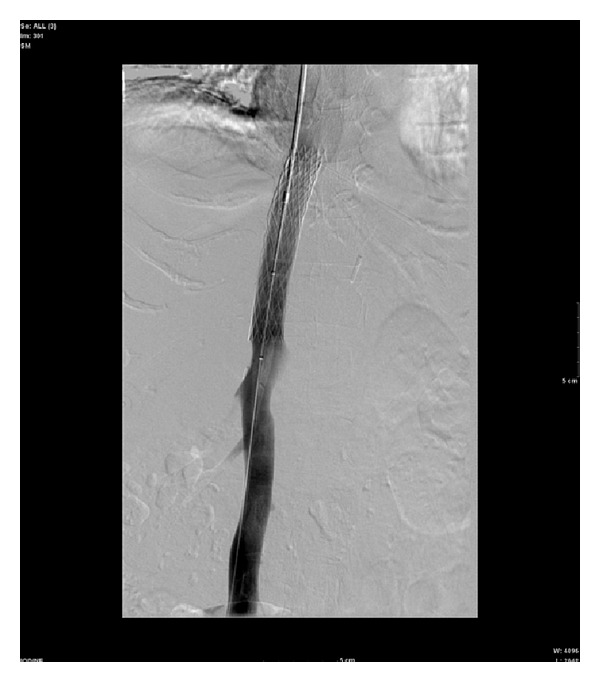


The patient showed minimal clinical improvement and underwent further inferior vena cavagram on the 35th day of admission revealing focal stenosis just distal to the two overlapping Palmaz stents. The gradient across the stenosis measured 12 mm Hg. The following day, a third 4010 Palmaz stent was mounted on a 16 mm diameter angioplasty balloon and deployed. Completion angiography demonstrated excellent angiographic result; however, the gradient remained 12 mm Hg (Figure 4).

Seven-minute delayed venogram revealed migration of stenosis distal to the third Palmaz stent compatible with torsion (Figure 5).

The patient was then taken for surgical decompression and revision 8 days later. The patient was found to have severe adhesions, which were lysed. Following surgical decompression of regional ascites with 8 liters of ascites evacuated, intraoperative ultrasound and venogram demonstrated resolution of stenosis and torsion. The patient's recurrent ascites, pleural effusions, and renal insufficiency resolved.

## 3. Discussion

Orthotopic liver transplantation (OLT) is a well-established method of treatment for end stage liver disease [1]. Outflow obstruction following OLT is a rare but serious complication with a reported incidence of 1–6% [2, 3]. Immediate OLT complications, such as torsion, are felt to be mostly technique related, while late OLT stenosis is most likely related to intimal hyperplasia or fibrosis at the anastomotic site [3, 4]. A significant gradient across the stenosis is defined as 7–10 mmHg [4, 5]. If left unrecognized or uncorrected, OLT outflow obstruction can lead to increased morbidity including decreased graft function, ascites, lower extremity edema, recurrent varices, decreased renal function, and/or decreased cardiac output [5–7].

Endovascular techniques are the preferred method for treating outflow obstruction/stenosis following OLT [4, 5]. Differentiating acute versus chronic stenosis should be considered for selecting the primary technique for treatment of the stenosis. For chronic stenosis, Guimarães et al. report that angioplasty alone is not sufficient to sustain patency of the vessel due to IVC elasticity and elastic recoil of the fibrotic tissue. In these cases, primary stenting may be warranted [4]. The consensus is to oversize the IVC stent by 2 mm to effectively treat the stenosis [4, 5]. Stenting has been proven to be effective in treatment of post-OLT venous stenosis [3, 4, 8–10].

The case we present was refractory to both angioplasty and stenting. Given the timing of the initial presentation of symptoms, the initial cause of the intermittent torsion was likely surgical technique related. We postulate that the increased pressure secondary to the regional ascites precluded continued patency of the IVC. The recurrent ascites, as well as the additional presenting symptoms, are felt to be initially secondary to intermittent IVC twisting/torsion following OLT. The initial ultrasound following the patient's presentation with shortness of breath was performed one day following paracentesis and did not demonstrate IVC stenosis. The ensuing ultrasound was performed 10 days following the most recent paracentesis and revealed severe IVC stenosis worse in the supine position. The time allowance for reaccumulation of fluid and change in the degree of stenosis with position suggests a pressure-related anomaly. It was hypothesized that, as hepatic decompensation progressed, the accumulation of fluid and pressure in the pericaval region in combination with development of severe adhesions resulted in entrapment of the torsion. The apparent resolution of the stenosis following stent placement with delayed migration caudally would be the result of the increased pressure. The stenosis was refractory to percutaneous drainage and resolved only with surgical lysis of adhesions and decompression. Surgical decompression is currently the treatment modality of choice for compressive ascites [11]. Compressive ascites resulting in IVC stenosis is very rare and the underlying cause remains unknown [11].

## Figures and Tables

**Figure 5:**